# Caffeine Inhibits Choroidal Neovascularization Through Mitigation of Inflammatory and Angiogenesis Activities

**DOI:** 10.3389/fcell.2021.737426

**Published:** 2021-10-14

**Authors:** Christine M. Sorenson, Yong-Seok Song, Ismail S. Zaitoun, Shoujian Wang, Barbara A. Hanna, Soesiawati R. Darjatmoko, Zafer Gurel, Debra L. Fisk, Colleen M. McDowell, Ryan M. McAdams, Nader Sheibani

**Affiliations:** ^1^Department of Pediatrics, University of Wisconsin School of Medicine and Public Health, Madison, WI, United States; ^2^McPherson Eye Research Institute, University of Wisconsin School of Medicine and Public Health, Madison, WI, United States; ^3^Department of Ophthalmology and Visual Sciences, University of Wisconsin School of Medicine and Public Health, Madison, WI, United States; ^4^Department of Human Oncology, University of Wisconsin School of Medicine and Public Health, Madison, WI, United States; ^5^Department of Cell and Regenerative Biology, University of Wisconsin School of Medicine and Public Health, Madison, WI, United States; ^6^Department of Biomedical Engineering, University of Wisconsin School of Medicine and Public Health, Madison, WI, United States

**Keywords:** angiogenesis, adenosine receptors, age-related macular degeneration, retinal and choroidal endothelial cells, choroidal melanocytes

## Abstract

Adenosine receptors (AR) are widely expressed in a variety of tissues including the retina and brain. They are involved in adenosine-mediated immune responses underlying the onset and progression of neurodegenerative diseases. The expression of AR has been previously demonstrated in some retinal cells including endothelial cells and retinal pigment epithelial cells, but their expression in the choroid and choroidal cells remains unknown. Caffeine is a widely consumed AR antagonist that can influence inflammation and vascular cell function. It has established roles in the treatment of neonatal sleep apnea, acute migraine, and post lumbar puncture headache as well as the neurodegenerative diseases such as Parkinson and Alzheimer. More recently, AR antagonism with caffeine has been shown to protect preterm infants from ischemic retinopathy and retinal neovascularization. However, whether caffeine impacts the development and progression of ocular age-related diseases including neovascular age-related macular degermation remains unknown. Here, we examined the expression of AR in retinal and choroidal tissues and cells. We showed that antagonism of AR with caffeine or istradefylline decreased sprouting of thoracic aorta and choroid/retinal pigment epithelium explants in *ex vivo* cultures, consistent with caffeine’s ability to inhibit endothelial cell migration in culture. *In vivo* studies also demonstrated the efficacy of caffeine in inhibition of choroidal neovascularization and mononuclear phagocyte recruitment to the laser lesion sites. Istradefylline, a specific AR 2A antagonist, also decreased choroidal neovascularization. Collectively, our studies demonstrate an important role for expression of AR in the choroid whose antagonism mitigate choroidal inflammatory and angiogenesis activities.

## Introduction

Fenestrated choroidal endothelial cells (ChEC) of the choriocapillaris aid in efficient transport of nutrients to the retinal pigment epithelium (RPE) and photoreceptor cells (Fields et al., 2020). ChEC also respond to pro-angiogenic and pro-survival growth factors including vascular endothelial growth factor (VEGF) with important roles in their survival and function. The choriocapillaris, which lies beneath the RPE, has the highest flow rate in the body (Pemp and Schmetterer, 2008) and plays an important role in vision health. Maintenance of choroidal homeostasis facilitates photoreceptor and outer retinal integrity, and function. The choroidal homeostasis is maintained by a balanced production of pro- and anti- angiogenic and inflammatory factors whose disruption contributes to the pathogenesis of sight-threatening eye diseases including exudative/neovascular age-related macular degeneration (nAMD) (Farnoodian et al., 2017). However, the mechanisms underlying these changes are not well-defined leaving gaps in our understanding of the most effective methods to treat choroidal neovascularization (CNV) in nAMD patients. VEGF is a major ocular proangiogenic factor with important roles in choroidal vascular homeostasis and is the target of current treatment for nAMD, although a significant portion of the patients are non-responsive (Kwak et al., 2000; Campochiaro and Akhlaq, 2020).

Retinal pigment epithelium is a major source of VEGF production with important roles in the health of choroid and retina (Saint-Geniez et al., 2009). Although VEGF expression is essential for RPE maintenance, its increased expression contributes to inflammation, CNV, and vessel leakiness. Anti-VEGF therapies such as bevacizumab (Avastin) and its closely related Fab fragment ranibizumab (Lucentis) are the current standard of care in the treatment of neovascular eye diseases (Hernandez-Zimbron et al., 2018). RPE atrophy is noted in about 20–30% of patients receiving anti-VEGF treatment (Munk et al., 2016). This further demonstrates the essential role VEGF plays in ocular tissue development and homeostasis (Carmeliet et al., 1996; Ferrara et al., 1996). Thus, in patients receiving anti-VEGF therapy for nAMD, the amount of VEGF needs to be reduced to prevent vascular inflammation, excessive permeability, and pathologic neovascularization but not so low to cause RPE atrophy. Clinically, this is challenging to establish and maintain, which may also explain, at least in part, the incomplete response to anti-VEGF noted in 30% of patients with nAMD (Mantel et al., 2020; Maroñas et al., 2020).

Aberrant inflammation and angiogenesis contribute to the pathogenesis of many ocular diseases including nAMD (Potilinski et al., 2021; Zhang and Wong, 2021). One of the several factors that influence angiogenesis is changes in oxygenation. Changes in oxygenation can occur due to disease processes or environment. Stress, trauma, inflammation, and ischemia cause ATP catabolism and subsequent release of adenosine (Riksen et al., 2003; Ryzhov et al., 2007; Ouyang et al., 2013; Bowser et al., 2017; Peleli and Carlstrom, 2017; Santiago et al., 2020). Adenosine mediates its physiologic effects through interactions with receptors denoted A1, A2A, A2B, and A3 (Auchampach, 2007; Ryzhov et al., 2007; Chen et al., 2013; Ouyang et al., 2013). These receptors are expressed on macrophages, endothelial cells, neutrophils, fibroblasts, and other cell types (Hasko et al., 2007). The localization and signaling pathways of adenosine receptors (AR) in various tissues including the retina have been the subject of numerous studies (Santiago et al., 2020). However, the cellular expression and signaling pathways of these receptors in the choroid remains largely unknown. Adenosine signaling through its receptors regulates inflammation and angiogenesis activities (Hasko and Cronstein, 2004; Montesinos et al., 2004; Ouyang et al., 2013). Thus, modulation of AR has been sought for the treatment of several diseases with inflammatory and/or neovascular components such as arthritis, cancer, diabetes, and wound healing (Montesinos et al., 2004; Lopez et al., 2018). There is compelling evidence for the therapeutic potential of AR antagonists in the treatment of ocular diseases characterized by inflammation and neovascularization (Santiago et al., 2020), like nAMD.

Caffeine is a widely consumed AR antagonist that exerts pharmacological effects on various organ systems (Cronstein, 2010; Dervişoğulları et al., 2016; Madeira et al., 2016, 2017; Kolahdouzan and Hamadeh, 2017; Zhang S. et al., 2017; Yoon and Danesh-Meyer, 2019). It is routinely given to preterm infants to treat apnea of prematurity, which also reduces oxygen-induced retinopathy and liver fibrosis (Cronstein, 2010; Zhang S. et al., 2017). The FDA lists caffeine as generally safe for human consumption, with 3–6 mg/kg representing the optimal dose for most people. The consumption of a single cup of coffee (Vural et al., 2014) or a single 200-mg caffeine capsule (Altinkaynak et al., 2016) in healthy subjects transiently decreases the choroidal thickness by 4 h. This decrease was attributed mainly to the vasoconstrictive effect of caffeine, but its chronic consequences on human health and morbidity remains unknown. In a prospective cohort from the Beaver Dam study (average follow-up of 4.8 years), coffee and caffeine consumption were not associated with an increased incidence of early age-related maculopathy, drusen deposits, or pigmentary abnormalities (Tomany et al., 2001). However, coffee consumption has been associated with increased risk for heart disease due to increased cholesterol and blood pressure (Boia et al., 2016). The mechanism by which caffeine mediates its various effects remains largely unknown and is further compounded by research findings that are frequently contradictory. A recent study by Stevens et al. showed increasing coffee consumption was associated with reduced long-term risk of heart failure in participants of the Farmington Heart Study (Stevens et al., 2021).

The protective effects of caffeine on the retina and lens have been the subject of many recent investigations (Yoon and Danesh-Meyer, 2019). Unfortunately, the relationship between caffeine and the eye is complicated by large inter-individual variability. These variables include pharmacological and health effects of caffeine, difficulty estimating dietary exposure to caffeine, and incomplete understanding of the pathological processes in the eye. The relationship between caffeine and the eye is particularly an issue in patients with other diseases associated with aging such as glaucoma and AMD (Santiago et al., 2020). Inflammation contributes to the normal aging, in general, and that of the retina (Steinle et al., 2009), and is a recognized target of the protective action of caffeine (Madeira et al., 2017). Here we addressed major gaps in our understanding of caffeine action in the retinal and choroidal vasculature by examining AR expression and the impact of their antagonism on sprouting angiogenesis and choroidal neovascularization.

## Materials and Methods

### Ethics Statement, Experimental Animals, and Cell Cultures

All animal experiments were carried out in accordance with and were approved by the Institutional Animal Care and Use Committee of the University of Wisconsin School of Medicine and Public Health. Immorto mice expressing a temperature-sensitive SV40 large T antigen were obtained from Charles River Laboratories (Wilmington, MA). These mice were back-crossed for at least 10 generations to C57BL/6J mice. Using these mice, we have reported the successful isolation and propagation of various ocular cell types including retinal endothelial cells (REC), retinal pericytes (RPC), retinal astrocytes (RAC), retinal pigment epithelium (RPE) cells, choroidal EC (ChEC), choroidal pericytes (ChPC), choroidal melanocytes (ChMC), and microglial cells (Su et al., 2003; Scheef et al., 2005, 2009; Fei et al., 2014; Farnoodian et al., 2015; Lavine et al., 2017). The identity of these cells has been previously confirmed by expression of specific cell markers, and their growth conditions are well detailed. Multiple isolations of these cells are available in the laboratory and were used in experiments described here. Human retinal endothelial cells were obtained from Cell Systems (Kirkland, WA) and ARPE19 cells were obtained from ATCC (Manassas, VA) and cultured under conditions as recommended by the suppliers. Deidentified human donor eyes were purchased from the Lions Eye Bank of Wisconsin (Madison, WI) and were used for the preparation of RNA from retina and choroid, and frozen sections for immune staining.

### Cell Viability Assays

To determine potential adverse effects of caffeine on various cells incubated with the different concentrations of caffeine, their viability was assessed after 48 h using an MTS cell viability assay kit (Promega, Madison, WI, United States; G5421) as recommended by the supplier. Various ocular cell types were plated at 1 × 10^4^ cells/well of a 96-well tissue culture plate in 0.1 ml of their corresponding growth medium overnight. The next day, each well received their appropriate medium containing different concentrations of caffeine (0, 100, 200, 400, 800, and 1,600 μM) and incubated for 48 h. The stock solution of caffeine citrate was obtained from our hospital pharmacy (caffeine citrate 20 mg/ml prepared in 5% dextrose: Exela Pharma Sciences, Lenoir, NC, United States). Following incubation, the percent cell viability relative to control (no caffeine; 5% dextrose) was determined as recommended by the supplier. Each group was done in triplicate and repeated with at least two different isolations of desired cell lines.

### Scratch Wound Assays

Endothelial cells (4 × 10^5^) were allowed to reach confluence (2–3 days) on a 60-mm dish. One day prior to wounding, the plates were incubated with vehicle, caffeine, or istradefylline at the desired concentrations noted in the figure legends. A wound was inflected to the cell layer with a 1-ml micropipette tip. The plates were rinsed and growth medium containing 5-fluorouracil (100 ng/ml, Sigma, St Louis, MO, United States; F6627) added to rule out the potential contribution of cell proliferation rate differences. The medium also contained the concentrations of vehicle, caffeine, or istradefylline (MedChemExpress, Monmouth Junction, NJ, United States; HY-10888- 10 mM stock in DMSO) as noted in the figure legend. Photographs were taken daily up to 72 h and migration assessed as percent of total distance covered as previously described (Grutzmacher et al., 2010). Each assay was performed in triplicate and repeated with at least two different isolation of cells.

### Aortic Ring *ex vivo* Sprouting Angiogenesis Assays

Periaortic fibro-adipose tissue was removed from the thoracic aortas of 3-week-old male and female C57BL/6j mice as we previously noted (Grutzmacher et al., 2010). Aortas were sectioned into 1-mm-long aortic rings with eight aortic rings per 35-mm dish. The Matrigel (BD Biosciences, San Diego, CA, United States; 356235; 0.5 ml of 10 mg/ml) coated dishes were incubated in a 37°C incubator for 30 min to harden. DMEM with 1% fetal bovine serum (FBS; 2 ml) was added to each dish, and the rings were allowed to attach overnight. The next day, the dishes were fed with fresh medium containing the corresponding vehicle, 200 μM caffeine or 10 μM istradefylline (diluted in 0.3% Tween 80, 10% sucrose in saline). The cultures were fed every other day and at 7 days were photographed using a Nikon microscope equipped with a digital camera. We determined the area of sprouting per mm of tissue using Image J software (NIH).^1^

### Choroid-Retinal Pigment Epithelium Complex *ex vivo* Sprouting Angiogenesis

Choroid-RPE was dissected from C57BL/6j male and female 3-week-old mice and sectioned into 0.5- to 1-mm pieces as we previously described (Farnoodian et al., 2015). The 10 pieces per eye were placed into 35−mm culture dish coated with 0.5 ml of Matrigel (10 mg/ml; BD Biosciences) and allowed to harden (30 min at 37^*o*^C). Endothelial cell growth medium (DMEM containing 10% FBS, 2 mmol/L L−glutamine, 2 mmol/L sodium pyruvate, 20 mmol/L HEPES, 1% non-essential amino acids, 100 μg/ml streptomycin, 100 U/ml penicillin, 55 U/ml heparin, and endothelial growth supplement 100 μg/ml (Sigma, St. Louis, MO, United States; E2759) was then added. After 48 h, the explants were fed every other day with the appropriate vehicle, 400 μM caffeine or 10 μM istradefylline. At 8 days, the explants were fixed with 4% paraformaldehyde and photographed (PFA; Electron Microscopy Sciences, Hatfield, PA, United States; 15710). The area of sprouting was quantified as previously described (Farnoodian et al., 2015).

### Laser Induced Choroidal Neovascularization

Male and female pubertal (8-week-old) or mature adult (4-month-old) C57BL/6j mice were given the appropriate dose of vehicle, caffeine, or istradefylline by gavage as delineated below. The day of laser photocoagulation, the mice were anesthetized with ketamine hydrochloride (80 mg/kg) and xylazine (10 mg/kg). To dilate the pupil, Tropicamide (1%) was used. A slit lamp delivery system of an OcuLight GL diode laser (Iridex, Mountain View, CA) located the 9, 12, and 3 o’clock positions of each eye (posterior pole) for laser photocoagulation (75 μm spot size, 0.1 s duration, 120 mW) with a handheld cover slip that served as a contact lens for viewing of the retina. Photocoagulation facilitated rupture of the Bruch’s membrane. To assess neovascularization the choroid-RPE complex was harvested 2 weeks post-laser and fixed in 4% PFA. The complex was incubated with blocking buffer (20% normal goat serum and 5% fetal calf serum in 1 × PBS) for 1 h followed by incubation with anti-ICAM-2 (BD Biosciences; 553326; 1:500 in 1 × PBS with 20% normal goat serum and 20% fetal calf serum) at 4°C overnight. Staining mononuclear phagocytes in choroid-RPE with anti-F4/80 (eBiosciences; 14-4801-82; 1:500 in 1 × PBS) was done as above with choroid-RPE harvested 6 days following laser photocoagulation. Following tissue washing, the complex was incubated with the appropriate secondary antibody and images were captured with a Zeiss microscope (Zeiss, Chester, VA, United States) in digital format. The total area (in μm^2^) of each individual CNV or mononuclear phagocyte recruitment area was measured using Image J software (National Institute of Mental (NIH; see text footnote 1).

Treatment with caffeine or istradefylline was as follows: 9 days before laser photocoagulation (day 9) male and female C57BL/6j mice were given caffeine citrate 20 mg/kg (in 5% dextrose) or vehicle (5% dextrose) by gavage (10 mice for each group). Subsequent doses of caffeine citrate were 10 mg/kg. Caffeine or vehicle was administered the next 4 days (day 8 to 5). Then, the mice received caffeine or vehicle at days 1 and2 (excluding weekends) prior to laser. Following laser photocoagulation, the mice received caffeine or vehicle by gavage daily excluding weekends for the next 2 weeks. Mice received istradefylline 3 mg/kg (in 0.3% Tween 80, 10% sucrose in saline) or vehicle by gavage using the same dosing times prior and following laser photocoagulation. For regression studies, the mice received caffeine by gavage at 20 mg/kg on day 5 following laser photocoagulation and 10 mg/kg caffeine until choroid-RPE harvest at 2 weeks post-laser photocoagulation. Areas of neovascularization were assessed as described above.

### RNA Purification and Real-Time qPCR Analysis

Retinas and choroidal/RPE tissues, as well as various cell types, were lysed and homogenized in Trizol (Invitrogen, San Diego, CA, United States) reagent. Total RNA was extracted using RNeasy mini kit (Qiagen, Maryland, CA, United States) and 1 μg total RNA was used for cDNA synthesis with an RNA to cDNA EcoDry Premix (TaKaRa, Mountain View, CA, United States). For qPCR, cDNA (1 μl each diluted 1:10) was used, and assays were performed in triplicate of three biological replicates using the Mastercycler Realplex (Eppendorf) and TB-Green advantage qPCR Premix (TakaRa). Amplification parameters were as follows: 95°C for 2 min; 40 cycles of amplification (95°C for 15 s and 60°C for 40 s); dissociation curve step (95°C for 15 s, 60°C for 15 s, 95°C for 15 s). We used the primers noted in Table 1. Standard curves were generated from known quantities of each target gene with linearized plasmid DNA. We used 10 times dilution series for each known target, which we amplified using TB-Green qPCR. The linear regression line for DNA (ng) was assessed from relative fluorescent units (RFU) at a threshold fluorescence value (Ct). Gene targets were quantified from cell extracts by comparing the RFU at the Ct to the standard curve and normalized by the simultaneous amplification of RpL13a, a housekeeping gene.

**TABLE 1:** Primer sequences.

	Forward 5′ to 3′	Reverse 5′ to 3′
Adora1	GTCAAGATCCCTCTCCGGTA	CAAGGGAGAGAATCCAGCAG
Adora2a	GGTCCTCACGCAGAGTTCC	TCACCAAGCCATTGTACCG
Adora2b	CCGATATCTGGCCATTCG	AGTCAATCCAATGCCAAAGG
Adora3	CTCTTTGCTAGGATTGCTTGG	AGAAGGAATGCCAAGAGCAG
Ccl11	CTGCTCACGGTCACTTCCTT	TGGGGATCTTCTTACTGGTCA
Ctgf	TCCCGAGAAGGGTCAAGCT	TCCTTGGGCTCGTCACACA
Cx3cl1	CGCGTTCTTCCATTTGTGTA CTC	GCACATGATTTCGCATTTCG
Cxcl1 (Kc)	ACAGGGGCGCCTATCGCCAA	CGGTTTGGGTGCAGTGGGC
Cxcl10	TGGCTGTCCTAGCTCTGT ACTGT	GAGGACAAGGAGGGTGTGG
Cxcl2 (Mip2)	CCCTTGGACATTTTATGTC TTCC	GACACGAAAAGGCATGACAA
Icam1	GCCATAAAACTCAAGGGACAA	GGCTGAGGGTAAATGCTGTC
Il-18	AAGAAAATGGAGACCTGGA ATCAG	ATTCCGTATTACTGCGGTTG TACA
Il-1b	GTTCCCATTAGACAACTGCACT	CCGACAGCACGAGGCTTTT
Il-6	CAACCACGGCCTTCCCTACT	TTGGGAGTGGTATCCTCT GTGA
MCP-1	GTCTGTGCTGACCCCAAGAAG	TGGTTCCGATCCAGGTTTTTA
Pedf	GCCCAGATGAAAGGGAAGATT	TGAGGGCACTGGGCATTT
Stat3	ACCAACATCCTGGTGTCTCC	CACTACCTGGGTCGGCTTC
Thbs1	TGGCCAGCGTTGCCA	TCTGCAGCACCCCCTGAA
Tnfa	ACCGTCAGCCGATTTGCTAT	TTGACGGCAGAGAGGAGGTT
Vcam1	TCGCGGTCTTGGGAGCCTCA	TGACTCGCAGCCCGTAGTGC
Vegf	GGAGAGCAGAAGTCCCATGA	ACTCCAAGGGCTTCATCGTTA
RpL13a	TCTCAAGGTTGTTCG GCTGAA	CCAGACGCCCCAGGTA
**Human primers**		
ADORA1	GTCCGGTCCTCATCCTCAC	CCACCATCTTGTACCGGAGA
ADORA2A	CACACCACTCTCCCTAGA CTCTC	TTCCTCACACTTACATTTT TCCTG
ADORA2B	CACTGCTTATAATGCTGGTG ATCTA	GGGTGGTCCTCGAGTGGT
RPL13A	AAGCGGATGAACACCAACC	TGTGGGGCAGCATACCTC

*Ctgf, connective tissue growth factor; Pedf, pigment epithelium derived factor; Stat3, transcription3; Thbs1, thrombospondin-1; Tnf-a, tumor necrosis factor-a; Vegf, vascular endothelial growth factor.*

### Statistical Analysis

The differences between groups were evaluated with a one-way ANOVA and Dunnett’s multiple comparison test using Prism 8.0 (GraphPad). The comparison between two samples was confirmed with a *t*-test (paired or unpaired) or non-parametric tests (Mann–Whitney *U*) depending on whether the data followed a Gaussian distribution as determined by normality tests (D’Agostino and Pearson). Mean ± standard deviation is shown. *p* < 0.05 was considered significant.

## Results

### Adenosine Receptor Expression in Various Ocular Cells, Retina, and Choroid/Retinal Pigment Epithelium Tissues

**FIGURE 1:**
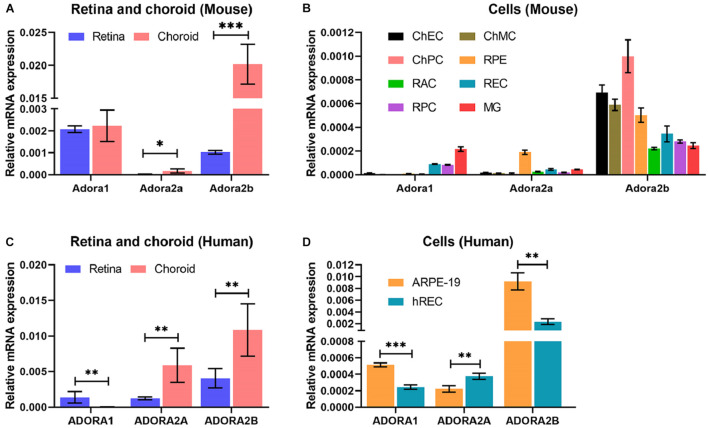
Adenosine receptor expression in ocular cells and tissues. Expression of adenosine receptors (AR), A_1_ (Adora1), A_2__*A*_ (Adora2a), A_2__*B*_ (Adora2b), and A_3_ (Adora3) in murine ocular tissues **(A)** and cells **(B)** prepared from 2-month-old mice (tissues) and 1-month-old mice (cells) and human ocular tissues **(C)** and cells **(D)** were determined by qPCR. These included retinal and choroid/RPE tissues **(A)** and retinal microglia (MG), retinal astrocytes (RAC), choroidal endothelial cells (ChEC), choroidal pericytes (ChPC), choroidal melanocytes (ChMC), retinal endothelial cells (REC), retinal pericytes (RPC), and retinal pigment epithelium (RPE) cells. Please note highest A_1_ expression in microglia, A_2__*A*_ expression in RPE cells, and A_2__*B*_ in ChEC, ChPC, ChMC, and RPE cells. The A_3_ level was very low in all cells examined. The retina and choroid/RPE tissues showed higher A1 and A2B. A similar expression pattern was noted in human tissues **(C)**, and REC and RPE (ARPE19) cells. (*P < 0.05, **P < 0.01, ***P < 0.001).

The expression and impact of the various AR in retina and choroid, as well as ocular vascular and non-vascular cells has not been well studied. To gain an appreciation of their expression, we utilized retina or choroid/RPE tissues and various ocular cell types derived from mouse retina and choroid (Figure 1). Adora1 and Adora2b were expressed in choroid/RPE, but their expression was more abundant in the retina, whereas Adora2a expression low in the retina, but was more prominent in the choroid/RPE. Adora2a expression was low in the retina, and Adora1 and Adora2b were expressed in choroid/RPE (Figure 1A). Consistent with the tissue expression patterns, Adora1 expression was mainly noted in microglia, retinal EC, and PC; minimal expression was observed in retinal AC, ChEC, ChPC, ChMC, and RPE cells. The expression of Adora2a was concentrated in RPE cells in contrast to Adora2b, which had more ubiquitous expression (highest in choroidal EC, PC, RPE, and MC). The level of Adora3 was the lowest of all the receptors examined with great variation among all samples (Figure 1B and Supplementary Figure 1). A similar pattern of AR expression was observed in human retina, choroid/RPE, retinal EC, and RPE cells (Figures 1C,D). Thus, AR were expressed to various degrees in all the ocular tissues and cell types examined, with prominent expression of Adora1 in microglia, Adora2a in RPE, and Adora2b in choroidal EC, PC, RPE, and MC, in similar patterns in both mouse and human samples. These results were further confirmed by immune staining of mouse and human eye sections using AR antibodies to A_1_, A_2__*A*_ and A_2__*B*_ (Supplementary Figure 2).

### Caffeine Exhibits Minimal Cytotoxicity in Various Ocular Cell Types

**FIGURE 2:**
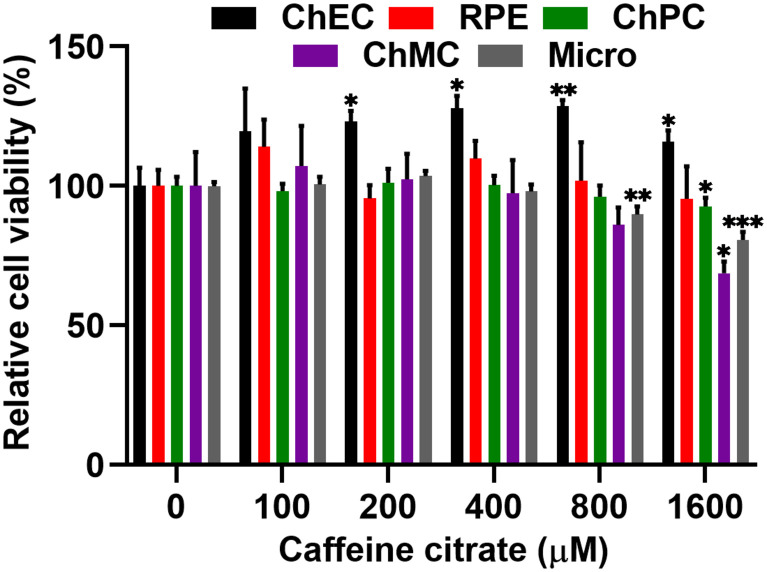
Minimal toxicity was noted in ocular cells incubated with different concentrations of caffeine. Cell viability relative to control cells, was assessed after 48 h of incubation with different concentrations of caffeine as detailed in Methods. Each experiment was done in triplicate and repeated with at least two different isolation of the desired cells (*p < 0.05, **p < 0.01, ***p < 0.001).

We examined the impact of various caffeine concentrations on viability of ChEC, ChPC, ChMC, RPE cells, and microglial cells. Figure 2 shows that caffeine up to 400 μM had no significant effect on the viability of these cells. We noted a significant increase in the viability of ChEC incubated with caffeine from 200 to 1,600 μM. However, caffeine at 800 μM resulted in a significant decrease in viability of microglial cells. Similarly, caffeine at 1,600 μM significantly reduced the viability of ChPC, ChMC, and microglial cells.

### Caffeine Inhibits Retinal and Choroidal Endothelial Cell Migration

**FIGURE 3:**
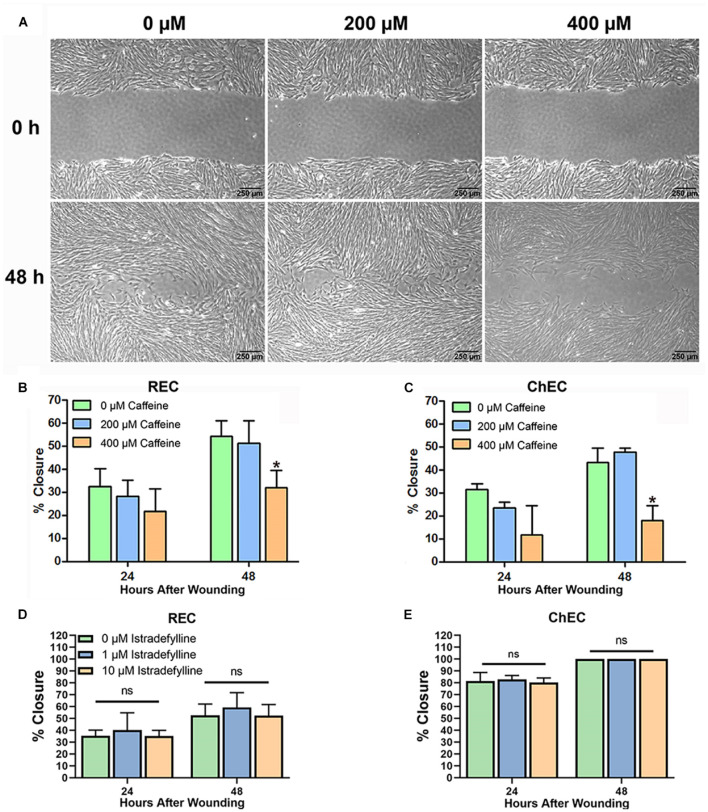
Caffeine mitigates choroidal and retinal endothelial cell migration. Migration of retinal endothelial cells (REC) and choroidal endothelial cells (ChEC) incubated with caffeine was determined using a scratch wound assay and monitored by phase microscopy. The wound images from ChEC are shown in panel **(A)**. The percent wound closure was quantified using ImageJ at 24 and 48 h compared with 0 h [**(B)** retinal EC; **(C)** ChEC]. Please note significant wound closure with vehicle while cells are incubated with 400 μM caffeine lagged behind. These caffeine concentrations had no effect on viability after 48 h (Figure 2). Incubation of REC **(D)** and ChEC **(E)** with 1 or 10 μM istradefylline did not significantly inhibit their migration. The treatments in panel **(E)** are identical to those in panel **(D)**. (*P < 0.05).

Optimal EC migration is an important component for the process of normal or pathological angiogenesis. Here, we assessed the impact caffeine, an AR antagonist, and istradefylline an adenosine A_2__*A*_ receptor antagonist, have on migration of retinal and choroidal EC (Figures 3A–C). Caffeine at 400 μM decreased retinal and choroidal EC migration, while 200 μM had no significant effect. Istradefylline did not significantly impact choroidal and retinal EC migration at 1 or 10 μM concentrations (Figures 3D,E).

### *Ex vivo* Choroid/Retinal Pigment Epithelium Explant Sprouting Angiogenesis Is Inhibited by Caffeine

**FIGURE 4:**
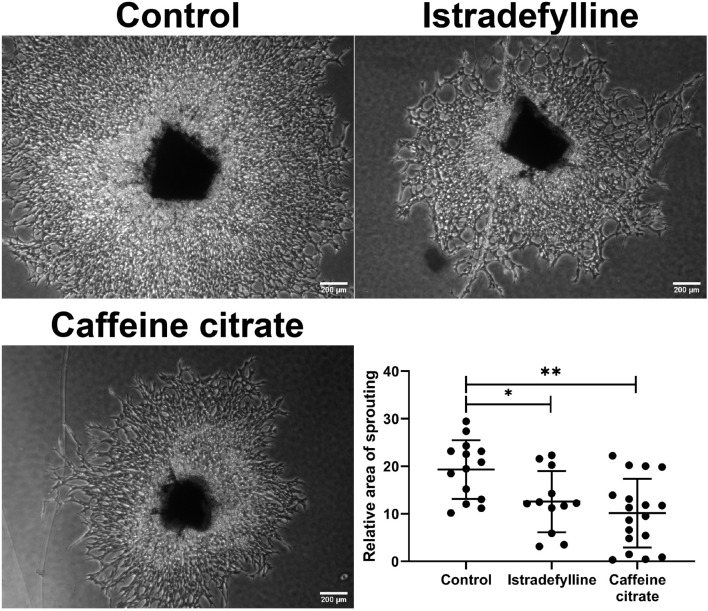
Caffeine mitigates choroid-retinal pigment epithelium (RPE) sprouting in *ex vivo* culture. Choroid/RPE complex isolated from 4-month-old C57BL/6J mice were grown for 7 days in the presence of vehicle (control), istradefylline (10 μM) or caffeine citrate (400 μM), and photographed. Image J was used to assess sprouting area. Scale bar = 200 μm (^∗^*p* < 0.05; ^∗∗^*p* < 0.01).

**FIGURE 5:**
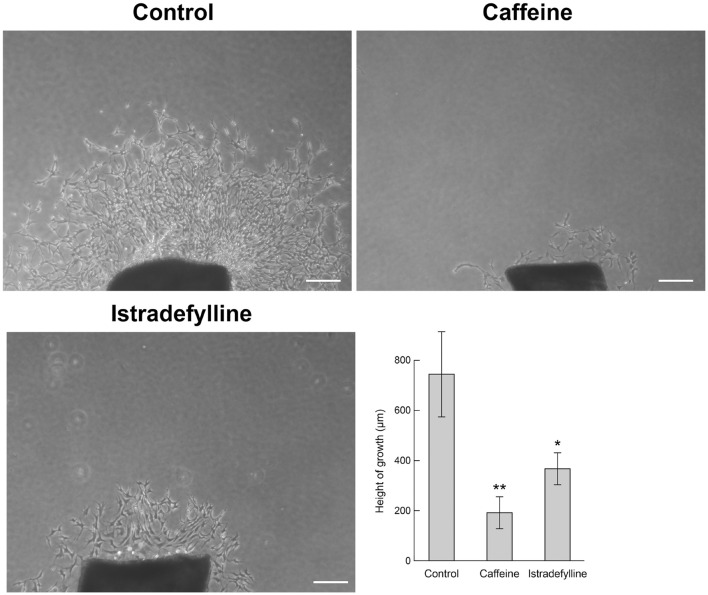
Caffeine and istradefylline mitigate thoracic aortic ring sprouting. Aortic rings were prepared from 3-week-old C57BL/6j mice, embedded in Matrigel in the presence of caffeine (200 μM), istradefylline (selective A_2__*A*_ receptor antagonist; 10 μM) or vehicle. Seven days later, aortic rings were photographed, and the mean area of outgrowths was quantified. Scale bar = 200 μm (^∗^*p* < 0.05; ^∗∗^*p* < 0.01).

To confirm changes in migration equated with altered sprouting capacity, we next used an *ex vivo* choroid/RPE sprouting assay. We have found the *ex vivo* choroid/RPE sprouting assay to be a very reproducible measure of sprouting angiogenesis, since it maintains the local interactions of EC with neighboring pericytes and RPE cells (Shao et al., 2013). We prepared choroid/RPE explants and assessed sprouting of *ex vivo* choroid/RPE cultures in the presence of vehicle, caffeine (400 μM), or istradefylline (10 μM) (Figure 4). Both caffeine and istradefylline decreased sprouting of choroid-RPE cultures, although to varying extents with caffeine demonstrating a more pronounced decrease in choroid-RPE sprouting. As an alternative method to choroid/RPE cultures to assess sprouting, we also utilized a thoracic aorta-sprouting assay. Thoracic aortas removed from C57BL/6j mice were embedded in Matrigel and incubated with vehicle, caffeine (200 μM), or istradefylline (10 μM) (Figure 5 and Supplementary Figure 3). Both caffeine and istradefylline inhibited sprouting of the embedded aortic rings. Thus, caffeine and istradefylline appear to effectively inhibit sprouting in *ex vivo* angiogenesis assays.

### Caffeine Impact on the Expression of Angioinflammatory Mediators of Retinal and Choroidal Endothelial Cells

**FIGURE 6:**
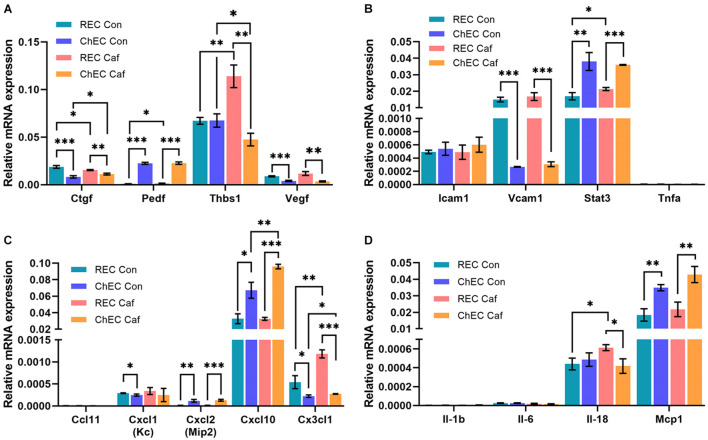
Expression of pro- and anti-angiogenesis and inflammatory mediators in retinal (REC) and choroidal endothelial cells (ChEC) incubated with caffeine. The expression levels of various factors that modulate angiogenesis and inflammation was assessed by qPCR analysis including **(A)** angiogenesis and inflammatory genes, **(B)** cell adhesion molecules **(C)** chemokines, and **(D)** cytokines. These experiments were done in triplicate and repeated with RNA from two different isolations retinal and choroidal EC (^∗^*p* < 0.05; ^∗∗^*p* < 0.01; ^∗∗∗^*p* < 0.001). RpL13A, 60S ribosomal protein L13a was used as control. Please note a significant increase in thrombospondin-1 (Thbs1) in REC and its decreased expression in ChEC with caffeine. Cxcl10 level increased in ChEC incubated with caffeine. The level of Cx3Cl1 increased in both REC and ChEC. An increase in IL-18 levels was noted in the retina.

Next, we assessed whether caffeine modulated expression of genes known to impact angiogenesis and inflammation in choroidal and retinal EC. The choroidal and retinal EC were incubated with caffeine (400 μM) or vehicle (0.5% Dextrose) for 24 h. RNA was isolated and analyzed for expression of various angiogenesis and inflammatory mediators using specific primers (Table 1) by quantitative real time PCR. Figure 6 demonstrates that the expression levels of angiogenesis and inflammatory mediators vary between choroidal and retinal EC, and with caffeine treatment. ChEC expressed lower levels of connective tissue growth factor (Ctgf) than retinal EC and caffeine had a modest, but significant and opposite effect on the level of Ctgf, with expression decreased in retinal EC but increased in ChEC. Compared with retinal EC, ChEC expressed higher levels of pigment epithelium derived factor (Pedf). Caffeine had no significant effect on Pedf expression in ChEC. ChEC expressed similar levels of thrombospondin-1 (Thbs1) as retinal EC. However, Thbs1 level was significantly increased in retinal EC but deceased in ChEC with caffeine treatment. ChEC expressed lower VEGF levels compared with retinal EC; VEGF levels were unaffected by caffeine treatment.

ICAM-1, a cell adhesion molecule whose expression is very low on EC, but increases in response to inflammatory mediators, is involved in leukocyte endothelium adhesion and blood-to-tissue transmigration. ChEC and retinal EC showed similar expression of ICAM-1 which was not affected by caffeine. VCAM-1, a cell adhesion molecule that mediates the interaction of endothelial cells and perivascular supporting cells, was expressed at significantly higher levels in retinal EC compared with ChEC, but its expression was not significantly affected by caffeine. Signal transducer and activator of transcription 3 (Stat3) is a transcription factor whose increased expression is associated with enhanced inflammatory conditions. Although Stat3 expression was significantly higher in ChEC compared with retinal EC, its level was not affected by caffeine in ChEC and only modestly, but significantly, increased in retinal EC. Tumor necrosis factor-α (Tnf-α) level was very low in both retinal and choroidal EC with or without caffeine.

We also examined the expression of several cytokines known to impact the inflammatory state of the eye including Ccl11, Cxcl1, Cxcl2, Cxcl10, and Cx3cl1. The level of Ccl11 was undetectable. A similar expression of Cxcl1 was noted in retinal EC and ChEC that was not affected with caffeine treatment. However, its level was modestly, but significantly, lower in ChEC compared with retinal EC. Expression for Cxcl10 was the highest among the chemokines examined; compared with retinal EC, its level was significantly higher in ChEC, especially when incubated with caffeine. Caffeine had no significant effect on Cxcl10 expression in retinal EC. The Cx3cl level was significantly higher in retinal EC compared with ChEC, with levels further increased with caffeine. Caffeine had no effect on Cx3cl1 expression in ChEC. These cells expressed very low levels of IL-1β and IL-6 and were unaffected by caffeine. The level of IL-18 was similar in ChEC and retinal EC. Caffeine treatment caused a modest but significant increase in retinal EC IL-18 level with no effect on ChEC levels. The Mcp1 level was similarly expressed in these cells and significantly increased with caffeine.

### Caffeine Mitigates Choroidal Neovascularization *in vivo*

**FIGURE 7:**
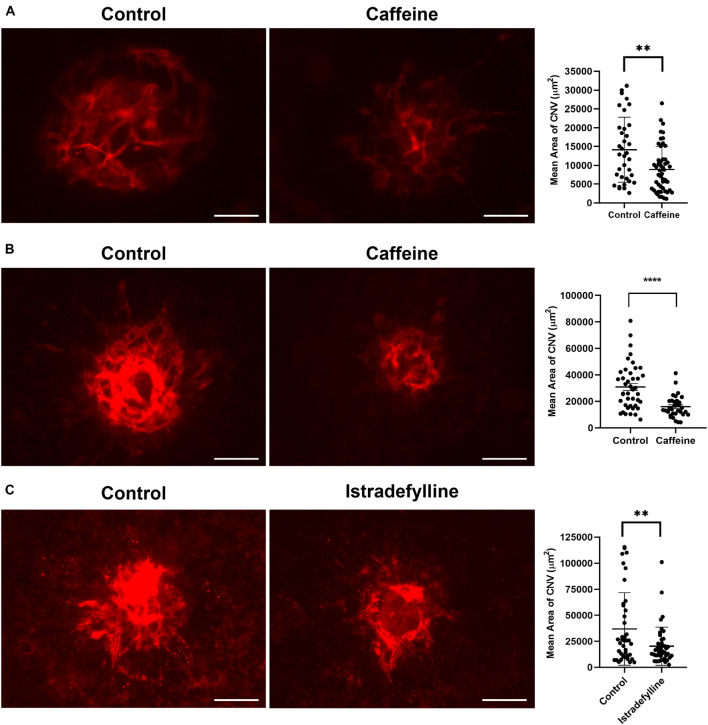
Caffeine mitigates choroidal neovascularization (CNV). The 2-month-old **(A)** and 4- month-old mice **(B)** (C57BL/6j male and female) were gavaged with caffeine 20 mg/kg first dose and 10 mg/kg subsequent doses or vehicle starting 5 days prior to laser. Treatments continued until the choroid/RPE was harvested 2 weeks later and stained with anti-ICAM-2. A significant mitigation of choroidal neovascularization (CNV) was noted in mice treated with istradefylline **(C)**. Scale bar = 200 μm (**p < 0.01, ****p < 0.0001; *n* = 10 mice/group).

The choriocapillaris nourishes the outer retina (Lutty et al., 2010). Dysfunction of the choriocapillaris contributes to pathogenesis of AMD and CNV (Biesemeier et al., 2014; Arya et al., 2018; Lutty et al., 2020). Given the ability of caffeine to inhibit migration of ChEC and choroid-RPE explant sprouting, we next determined its impact on CNV following laser photocoagulation-induced rupture of the Bruch’s membrane. Pubertal (2-month-old) or mature (4-month-old) adult C57BL/6j mice received vehicle or caffeine by gavage prior to and following laser photocoagulation-induced rupture of the Bruch’s membrane, as noted in Methods. As a quantitative measure of CNV, ICAM-2 staining was performed 2 weeks later. Caffeine treatment significantly reduced the degree of CNV in both pubertal and mature adult mice (Figures 7A,B).

Istradefylline inhibited *ex vivo* sprouting of choroid-RPE and thoracic aortic explants. We assessed whether istradefylline had a similar impact on pubertal mice treated with istradefylline (3 mg/kg) or vehicle by gavage prior to and following laser photocoagulation. Mice that received istradefylline demonstrated decreased area of CNV as noted by ICAM-2 staining (Figure 7C). Thus, our data indicate that antagonism of AR, at least in part through A_2__*A*_, is an effective means to mitigate CNV.

**FIGURE 8:**
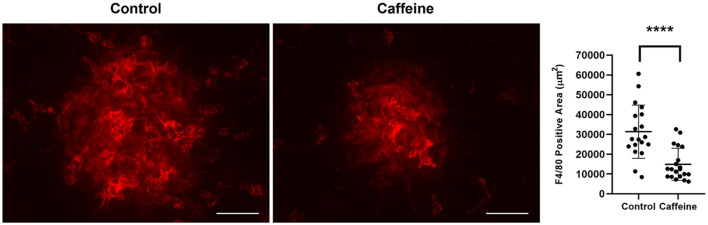
Caffeine treatment reduces macrophage accumulation. Mice (2-month-old C57BL/6j male and female) received caffeine 20 mg/kg first dose and 10 mg/kg subsequent doses or vehicle starting 5 days prior to laser by gavage. Treatments continued until RPE/choroid was harvested 6 days later and stained with anti-F4/80. The degree of macrophage recruitment was assessed as detailed in Methods. Scale bar = 200 μm (****p < 0.0001; *n* = 10 mice/group).

Next, we determined whether the decrease in CNV with caffeine administration resulted in a decrease in infiltration of mononuclear phagocytes. We have previously examined the temporal distribution of mononuclear phagocytes by staining choroid/RPE flat mounts prepared from mice at different times post laser. As we showed previously, by 3 days after laser, there was a significant accumulation of F4/80^+^ cells at sites of CNV lesions, which remained the same after 6 days post laser. However, by 14 days after laser, there was more than threefold decrease in the density of F4/80^+^ cells [(Wang et al., 2012b) and our unpublished data]. To determine the impact of caffeine on density of F4/80^+^ cells, we harvested choroid/RPE samples 6 days following laser photocoagulation from mice treated with vehicle or caffeine and stained with anti-F4/80. Figure 8 shows a significant decrease in the amounts of F4/80 staining in choroid-RPE samples from caffeine treated mice compared with vehicle-treated mice. Thus, caffeine mitigates the recruitment of mononuclear phagocytes to the laser photocoagulation site and correlates with decreased CNV.

### Caffeine Treatment Impact on Choroidal Neovascularization Regression

**FIGURE 9:**
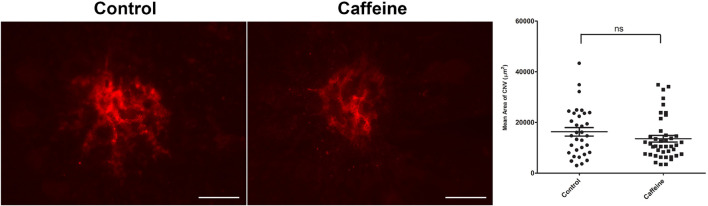
Caffeine is not effective when administered to animals with established choroidal neovascularization (CNV). Mice (2-month-old; C57BL/6j male and female) received caffeine 20 mg/kg first dose and 10 mg/kg subsequent doses or vehicle starting 5 days following laser photocoagulation. Treatments continued until the choroid/RPE was harvested 2 weeks following laser photocoagulation and stained with anti-ICAM-2. Scale bar = 200 μm (ns = not significant; *n* = 10 mice/group).

CNV begins forming shortly after laser photocoagulation. Here, we asked whether caffeine treatment could decrease the degree of CNV once establishment had begun, that is to promote CNV regression (Figure 9). Caffeine treatment was begun 5 days following laser photocoagulation. Although caffeine treated mice had a lower level of CNV compared with vehicle, this difference did not reach a significant level. Thus, caffeine treatment did not significantly mitigate further CNV formation or enhance CNV regression in these mice.

### The Impact of Laser and Age on Adenosine Receptor Expression in Retina and Choroid/Retinal Pigment Epithelium Tissues

**FIGURE 10:**
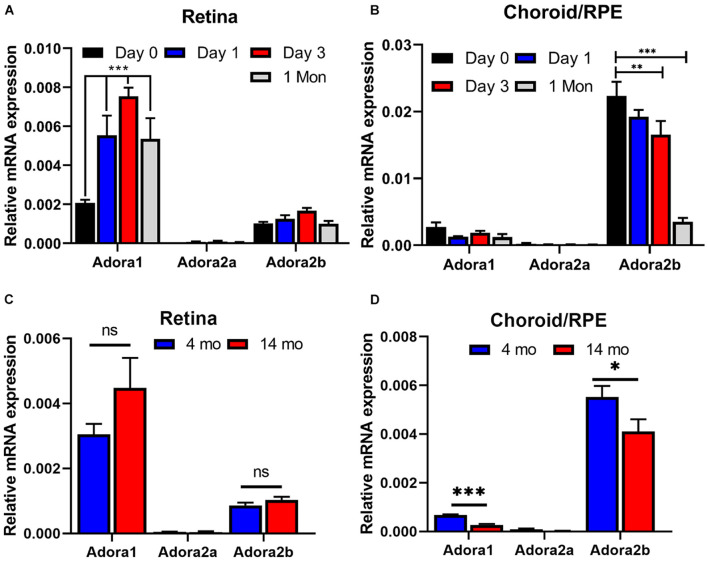
The impact of laser treatment and age on expression of adenosine receptors in retina and choroid. RNA was prepared from retina **(A,C)** and choroid/RPE **(B,D)** of mice [2-month-old **(A,B)**, 4-month- and 14-month-old **(C,D)**; C57BL/6j male and female] without laser or 1 day, 3 days, and 1 month post laser. Please note significant up regulation of A_1_ with laser treatment in the retina, while A_2__*B*_ was decreased in the choroid. Aging minimally affected adenosine receptor (AR) expression in the retina **(C)**. However, the expression of A_1_ and A_2__*B*_ was significantly decreased with age in choroid RPE **(D)**. (*p < 0.05, **p < 0.01, ***p < 0.001; *n* = 5 mice/group).

We next addressed whether expression of AR was influenced by laser treatment, and whether chronological age affects AR expression levels. RNA from retina or choroid/RPE samples were prepared from 2-month-old mice and examined for expression of various AR at different times post laser treatment. Figure 10A shows that in the retina, the expression of Adora1 receptor increases significantly a day after laser and stays high even after 1 month. Retinas expressed lower levels of Adora2a and Adora2b whose levels did not change after laser. In contrast, the choroid/RPE samples expressed similar levels of Adora1 receptor and low levels of Adora2a whose expression were not affected at different time post laser (Figure 10B). The expression of Adora2b is nearly 10-fold higher in choroid/RPE compared with the retina, and its expression significantly decreased (Ma and Wong, 2016) after laser in the choroid/RPE, especially after 1 month.

Since AMD pathogenesis and CNV is significantly impacted by aging (Chen et al., 2010; Sachdeva et al., 2014; Edwards et al., 2016; Ma and Wong, 2016), we determined whether the expression of adenosine receptors is affected in young compared with old animals. Retina and choroid/RPE RNA was prepared from 4-month- and 14-month-old mice and the AR levels were assessed by quantitative real time PCR. In the retina of young and old animals, the predominant AR was Adora1, followed by Adora2b (Figure 10C). The level of Adora2a was low, and the expression of these receptors was not significantly different between young and old mice. In the choroid/RPE the predominant receptor was Adora2b, followed by Adora1 and their expression was moderately, but significantly decreased in older animals (Figure 10D). The expression of Adora2a was very low in the choroid/RPE without any effect from aging. Thus, aging has a modest but significant effect on AR expression (Adora1 and Adora2b) in the choroid/RPE.

### Caffeine Treatment Does Not Influence Retinal Endothelial Cell or Pericyte Numbers in Developing Retinal Vasculature

The retinal vasculature forms in the mouse after birth with the superficial layer laid down through P7. This is followed by formation of the deep vascular plexus and intermediate layer, which are formed by P21. To assess whether caffeine treatment during retinal vascularization impacts retinal endothelial cell and pericyte numbers, C57BL/6j mice were administered caffeine or vehicle from P1–10 (intraperitoneal doses at P1, 20 mg/kg and P2–10 maintenance dose of 10 mg/kg caffeine or vehicle 5% dextrose). At P21, trypsin digestion of retinal preparations was performed, and the numbers of EC and PC were counted in blinded fashion (Table 2). No significant differences were noted in the number of EC and PC when mice were treated with caffeine compared with vehicle. Thus, caffeine treatment did not impact retinal vascular cell numbers in these studies.

**TABLE 2:** Retinal vascular cell density determined in trypsin digest whole mount retina preparations.

Vascular cells	Age	Control	Caffeine
Pericytes (PC)	P21	33.00 ± 4.30*	33.75 ± 2.63
Endothelial cells (EC)	P21	180.31 ± 20.56	169.06 ± 8.76

*Mean number of cells per high power field (×400).

*The p-values were calculated by comparing samples from control or caffeine treated mice at P21 and were not significant.*

## Discussion

Age-related macular degeneration is a major cause of vision impairment in the elderly. In its dry form (atrophic AMD) dysfunction and loss of retinal pigment epithelium leads to degeneration of photoreceptors and central vision impairment. The major loss of vision in AMD patients is associated with the wet/exudative form of the disease where growth of abnormal vessels into the retina, and its associated fibrosis and scar formation, lead to vision loss (Farnoodian et al., 2017; Handa et al., 2019). AMD is a multifactorial disease with aging as a major contributing factor. Inflammation is known to contribute to normal aging, and it is identified as an important factor in the pathogenesis of AMD. Adenosine, generated by catabolism of ATP is important in retinal vascular development and neovascularization, and modulation of inflammation and fibrosis (Afzal et al., 2003; Lutty and McLeod, 2003; Hua et al., 2007; Peng et al., 2008; Ryzhov et al., 2008; Zhang J. et al., 2017). The major activity of adenosine is mediated through ARs that are expressed on many cell types including vascular and inflammatory cells (Santiago et al., 2020). Excessive adenosine and AR-mediated signaling under hypoxic conditions contributes to ischemic retinopathy, and thus, are proposed to provide suitable target for therapeutic interventions (Lutty and McLeod, 2003; Zhou et al., 2018). Caffeine, a non-selective adenosine receptor antagonist, has shown effectiveness in mitigating retinal neovascularization (Chen et al., 2017; Zhang S. et al., 2017). In addition, a recent study demonstrated that endothelial expression of A_2__*A*_ is essential for pathological retinal neovascularization during oxygen-induced ischemic retinopathy (Liu et al., 2017). Furthermore, blockade of A_2__*A*_ in microglia diminishes inflammatory responses and protects RPE cell dysfunction and loss of photoreceptor cells in culture (Madeira et al., 2018). However, the contribution of adenosine and AR signaling to pathogenesis AMD, and whether their antagonism by caffeine mitigates CNV, remains unknown. Thus, given the important role of adenosine and its receptors in the regulation of inflammation, neovascularization and fibrosis, and their antagonism by caffeine, we proposed these receptor/ligand signaling axis could play a vital role in the pathogenesis of exudative AMD through modulation of angioinflammatory pathways, and can be mitigated by caffeine.

Adenosine stimulates the proangiogenic properties of retinal EC including proliferation, migration, and capillary morphogenesis. These activities are likely mediated through the A_2__*A*_ and/or A_2__*B*_ AR (Lutty et al., 1998; Afzal et al., 2003; Lutty and McLeod, 2003). The binding affinity to adenosine receptors for adenosine are A_1_ > A_3_ > A_2__*A*_ > A_2__*B*_, and for caffeine are A_2__*A*_ > A_1_ and A_2__*B*_ > A_3_ (nM for adenosine and μM for caffeine) (Kolahdouzan and Hamadeh, 2017). The mechanisms by which adenosine or its antagonist affect the angiogenic properties of retinal and choroidal neovascularization related cells remain unknown. We have established cultures of cells from retina and choroid with important roles in neovascularization. The current study demonstrates that these cells express AR namely A_1_, A_2__*A*_, A_2__*B*_, and A_3_ at varying levels. Although the AR signaling pathways have been previously studied in retinal vascular cells, further delineation is required in CNV-related cells (ChEC, ChPC, melanocytes, RPE cells, and microglia). We determined the impact caffeine has on migration and sprouting angiogenesis of CNV-related cells *in vitro* and *ex vivo*, and CNV *in vivo*. Understanding the response of CNV related cells to adenosine signaling, and its antagonism by caffeine, will allow us to delineate the cell autonomous mechanisms involved. This knowledge will facilitate the development of new therapeutics to circumvent the current incomplete therapy responses to anti-VEGF, and/or use for adjunct therapy.

In this study, choroidal related cells including ChEC, ChPC, ChMC, RPE, and microglial cells were used for all the experiments performed unless otherwise noted. Our laboratory routinely isolates murine choroidal and retinal related cells (Scheef et al., 2009; Fei et al., 2014), which were used here. These cells have been extensively characterized and shown to express all the appropriate cell type specific markers (Scheef et al., 2009; Fei et al., 2014). Using these cells, we showed their AR expression patterns, as well as in whole retina and choroid/RPE tissues. We showed A_1_ and A_2__*B*_ are predominantly expressed in the retina, while A_2__*A*_ is predominant in choroid/RPE with significant expression of A_1_ and A_2__*B*_. A_3_ levels were very low (near the limit of detection; not shown) and highly variable. Consistent with these expression patterns, we noted microglial cells express predominantly A_1_ with modest expression in retinal EC and PC. A_2__*A*_ expression was predominant in RPE cells with some expression in other cells from retina and choroid. A_2__*B*_ was more predominant in ChEC, ChPC, ChMC, and RPE cells, with some expression in other cells from retina and choroid. A_3_ expression was uniformly very low in all the cells from mouse retina and choroid. Given the fact that A_2__*A*_ is generally recognized as a caffeine receptor, which couples to stimulatory G proteins (Gα_*s/Olf*_) to activate adenylate cyclase and elevating cellular cAMP levels, we proposed its antagonism in the choroid/RPE could mitigate CNV through its anti-angiogenic and anti-inflammatory activities.

Adenosine stimulates the proangiogenic properties of retinal EC including proliferation, migration, and capillary morphogenesis. These activities were mediated through the A_2__*A*_ and/or A_2__*B*_ AR (Lutty et al., 1998; Afzal et al., 2003; Lutty and McLeod, 2003). The mechanism by which adenosine or its antagonist affects the angiogenic properties of CNV related cells remains unknown. To further confirm changes in the proangiogenic properties of CNV-related cells in response to caffeine, we examined the impact of caffeine and istradefylline on retinal and choroidal EC migration in culture. Although caffeine significantly reduced the migration of retinal and choroidal EC, istradefylline had no significant effect on the migration of these cells. This could be attributed to variation in AR expression patterns in these cells. It is also possible that higher concentrations of istradefylline might be needed. Future experiments will further address these possibilities.

We next used the *ex vivo* choroid/RPE and aorta explant sprouting assays to further address the impact of AR antagonism on sprouting angiogenesis. The choroid/RPE sprouting assay maintains local interactions of EC with neighboring cells (Shao et al., 2013). Our data demonstrated that decreased sprouting of choroid/RPE explants in the presence of caffeine and istradefylline, occurred at least in part through antagonism of A_2__*A*_ AR. However, the impact of caffeine was more significant than istradefylline, perhaps through antagonism of other AR receptors in addition to the A_2__*A*_, which is specifically targeted by istradefylline. Similar results were observed in the aortic sprouting assay. Thus, these effects of caffeine on the vasculature sprouting are not specific to retina and choroid.

Given the proposed anti-inflammatory and anti-angiogenesis activity of caffeine, we examined the expression of various genes with pro- and anti-angiogenesis and inflammatory activity that could be impacted by caffeine treatment. We noted a significant impact of caffeine on expression of Ctgf, Pedf, and Thbs1 in choroidal and retinal EC. Caffeine significantly decreased Ctgf expression in retinal EC, while it increased Pedf and Thbs1 expression. In contrast caffeine increased Ctgf expression, but decreased Thbs1 expression in ChEC, without affecting Pedf expression. The Vegf levels were lower in ChEC compared with retinal EC and caffeine had no effect on its expression. The increased Pedf and Thbs1 expression in retinal EC are consistent with the antimigratory effect of caffeine on retinal EC. We previously showed that Thbs1-deficiency results in enhanced proangiogenic activity of retinal EC, but not ChEC. Thbs1-deficient ChEC were less migratory compared with wild type cells (Wang et al., 2006; Fei et al., 2014). Thus, the decreased Thbs1 expression in ChEC is consistent with their reduced migration in the presence of caffeine (Wang et al., 2006; Fei et al., 2014; Falero-Perez et al., 2017; Farnoodian et al., 2018).

Although the expression of ICAM-1 and VCAM-1 were not affected by caffeine treatment, retinal EC expressed significantly higher levels of VCAM-1 compared with ChEC. This could be linked to the importance of EC and PC interactions in the retinal vasculature and development of blood-retina barrier (Garmy-Susini et al., 2005; Wang et al., 2012a). Among the chemokine examined Cxcl10 and Cx3cl1 were expressed at significantly higher levels and their expression was affected by caffeine treatment. The expression of Cxcl10 was significantly higher in ChEC compared with retinal EC, whose level was further increased with caffeine. The level of Cx3cl1 was significantly higher in retinal EC compared with ChEC, which was further increased with caffeine. The interaction of Cx3cl1 with its receptor Cx3cr1 on microglial cells plays an important role in neuroretina homeostasis and function (Raoul et al., 2010; Zhang et al., 2012, 2018). IL-18 was expressed at significantly higher levels than IL-1β, IL-6, and TNF-α in these cells, whose expression modestly, but significantly increased in retinal EC, but decreased in ChEC. The decreased level of IL-18 expression in ChEC with caffeine treatment is consistent with caffeine’s anti-inflammatory and protective activity in the choroid. The MCP-1 was expressed at significantly higher level in ChEC with no effect from caffeine on its expression in these cells. Collectively, caffeine treatment affects the expression of pro- and anti-angiogenic and inflammatory factors of the retina and choroid endothelium, which could contribute to the effect of caffeine in mitigating ocular inflammation and angiogenesis. A recent study identified tyrosinase as a major target of caffeine action in modulating the proliferation and expression of various immunomodulatory cytokines and chemokines in melanomas (Tabolacci et al., 2021). The role of choroidal melanocytes in immunomodulation and pathogenesis of nAMD, and their response to caffeine remain a subject of future investigation.

**FIGURE 11:**
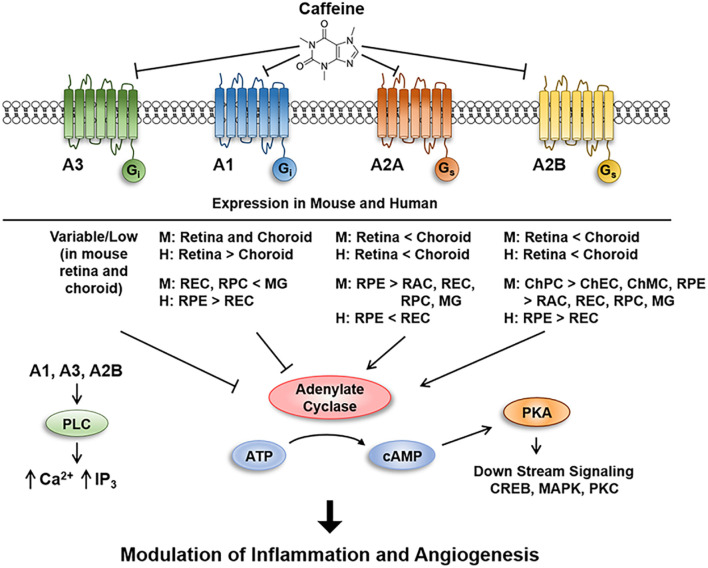
Schematic illustration of caffeine inhibition of signaling through adenosine receptors in retina and choroid. Inhibition of adenosine receptors mitigates sprouting angiogenesis *in vitro*, *ex vivo*, and *in vivo*, in part, by inhibiting the migration of endothelial cells through modulation of pro- and anti-angioinflammatory factors.

Caffeine and istradefylline were both efficacious in mitigating CNV, with caffeine being significantly more effective, as demonstrated in *ex vivo* sprouting assays. Mechanistically, this activity of caffeine was mediated by significantly attenuating the recruitment of inflammatory F4/80 + cells into the laser lesions. To determine how caffeine mediates these inhibitory effects requires further exploration to establish which cell types are involved and how they respond to caffeine. Here, we showed that microglial cells isolated from mouse retina predominantly express the A_1_ and A_2__*B*_, the receptors known as potential target of caffeine. We showed laser photocoagulation results in a significant upregulation of A_1_ receptor expression in the retina but not in the choroid, while A_2__*B*_ level decreases in the choroid, but not in the retina. Although aging had no significant impact on expression of A_1_ and A_2__*B*_ AR in the retina, their expression significantly decreased in choroid/RPE tissues. The decreased expression of these receptors in the choroid/RPE is consistent with increased inflammation noted with aging (Steinle et al., 2009; Xu et al., 2009; Ma and Wong, 2016). Thus, targeting of retinal microglial cells by caffeine through A_1_ AR may have significant impact on their phenotype preventing their migration to the sites of laser lesions, dampening the pro-inflammatory and angiogenesis activity in CNV lesions (summarized in Figure 11). Further delineation of AR involved in modulating murine choroidal inflammatory and angiogenesis activity will require conditional deletions of specific receptors in microglia or choroidal cells and assessment of the effect of caffeine in mitigating CNV. Identifying conditions that enhance AR expression with or without caffeine may provide further protection from CNV.

## Data Availability Statement

The original contributions presented in the study are included in the article/Supplementary Material, further inquiries can be directed to the corresponding author/s.

## Ethics Statement

The animal study was reviewed and approved by University of Wisconsin School of Medicine and Public Health Animal Use and Care Committee.

## Author Contributions

CS and NS were responsible for the experimental design, conducting experiments, protocol implementation, data analysis and interpretation, and writing and editing the manuscript and for the final approval of the article. Y-SS, IZ, SW, BH, SD, ZG, and DF were responsible for the experiments and data collection. CM and RM helped with the study design and editing of the manuscript. All authors read and approved the final manuscript.

## Conflict of Interest

The authors declare that the research was conducted in the absence of any commercial or financial relationships that could be construed as a potential conflict of interest.

## Publisher’s Note

All claims expressed in this article are solely those of the authors and do not necessarily represent those of their affiliated organizations, or those of the publisher, the editors and the reviewers. Any product that may be evaluated in this article, or claim that may be made by its manufacturer, is not guaranteed or endorsed by the publisher.

## Footnotes

1
http://rsb.info.nih.gov/ij
