# Supplementary material for: Caffeine Inhibits Choroidal Neovascularization Through Mitigation of Inflammatory and Angiogenesis Activities
Source: Front Cell Dev Biol. 2021 Oct 14;9:737426. doi: 10.3389/fcell.2021.737426 (PMC8551619; doi:10.3389/fcell.2021.737426)
Supplement: Supplementary file 1 [file Data_Sheet_1.PDF]

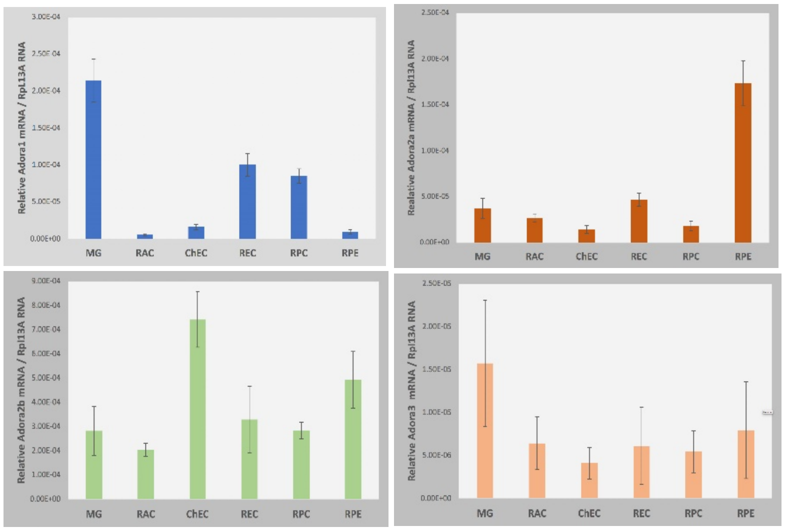

Supplementary Figure 1: Adenosine receptor expression in ocular cells. Expression of Adenosine receptors (<u>left to right</u>) A_1 (Adora1), A_{2A} (Adora2a), A_{2B} (Adora2b) and A_3 (Adora3) in murine ocular cells prepared from 3 week old mice was determined by qPCR. This included retinal microglia (MG), retinal astrocytes (RAC), choroidal endothelial cells (ChEC), retinal endothelial cells (REC), retinal pericytes (RPC) and retinal pigment epithelium (RPE) cells. Please note highest A_1 expression in microglia, A_{2A} expression in RPE cells, and A_{2B} in choroidal EC (ChEC). The A_3 level was relatively low in all cells.

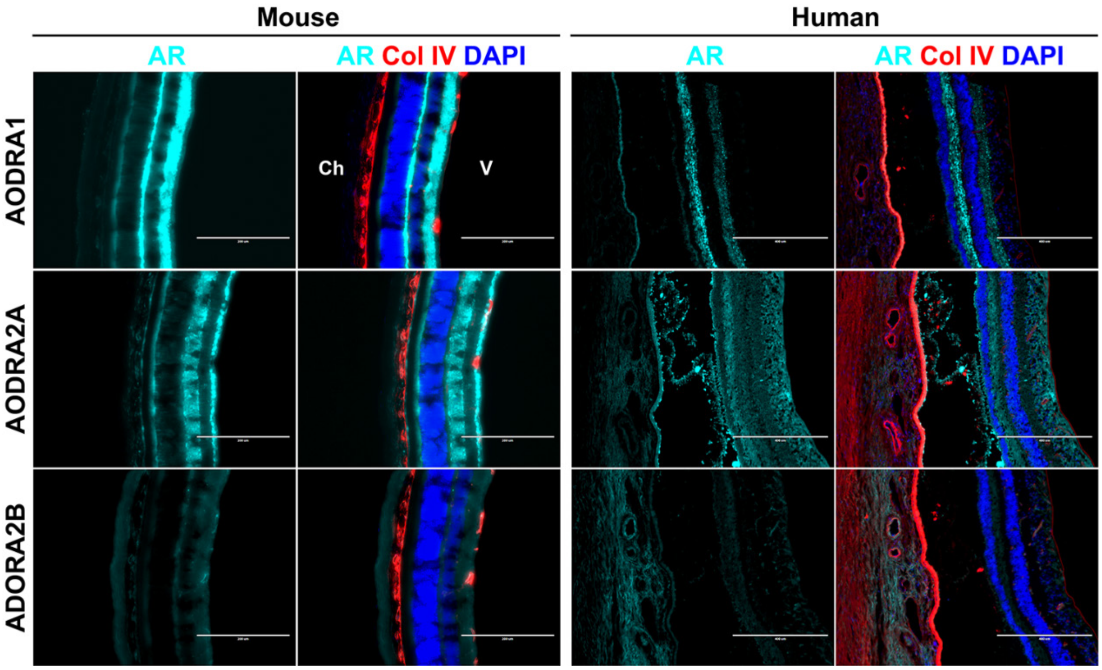

Supplementary Figure 2. Expression of AR in mouse and human eyes. Representative photomicrographs were taken from the cryosectioned adult mouse and human eyes. Sections were stained with antibodies against AR A1 (55026-i-AP), 2A (PA1-042) and 2B (PA5-72850) all from ThermoFisher and shown in cyan. Retinal and choroidal blood vessels were labelled with anti-Col IV (1340-01, SouthernBiotech) shown in red. All antibodies recognized both human and mouse targeted proteins. Secondary antibodies alone were used as negative control. The expression patterns for A1 (IOL and OPL); 2A (GCL, INL, Photoreceptor OS, RPE layer and choroidal blood vessels), and 2B (*brightest expression is in the retinal and choroidal blood vessels*). Less expression was observed in GCL, IPL, OPL and RPE. Please note that AR have similar expression patterns in mouse and human eyes. Scale bar: mouse images, 200 µm; human images, 400 µm. **V**: Vitreous; **Ch**: Choroid

Supplementary Figure 3. Aortic Sprouting was performed as detailed in Methods. For Quantitative assessment of sprouting four images (top, bottom, right and left from each explant) were captured and used for the analysis.

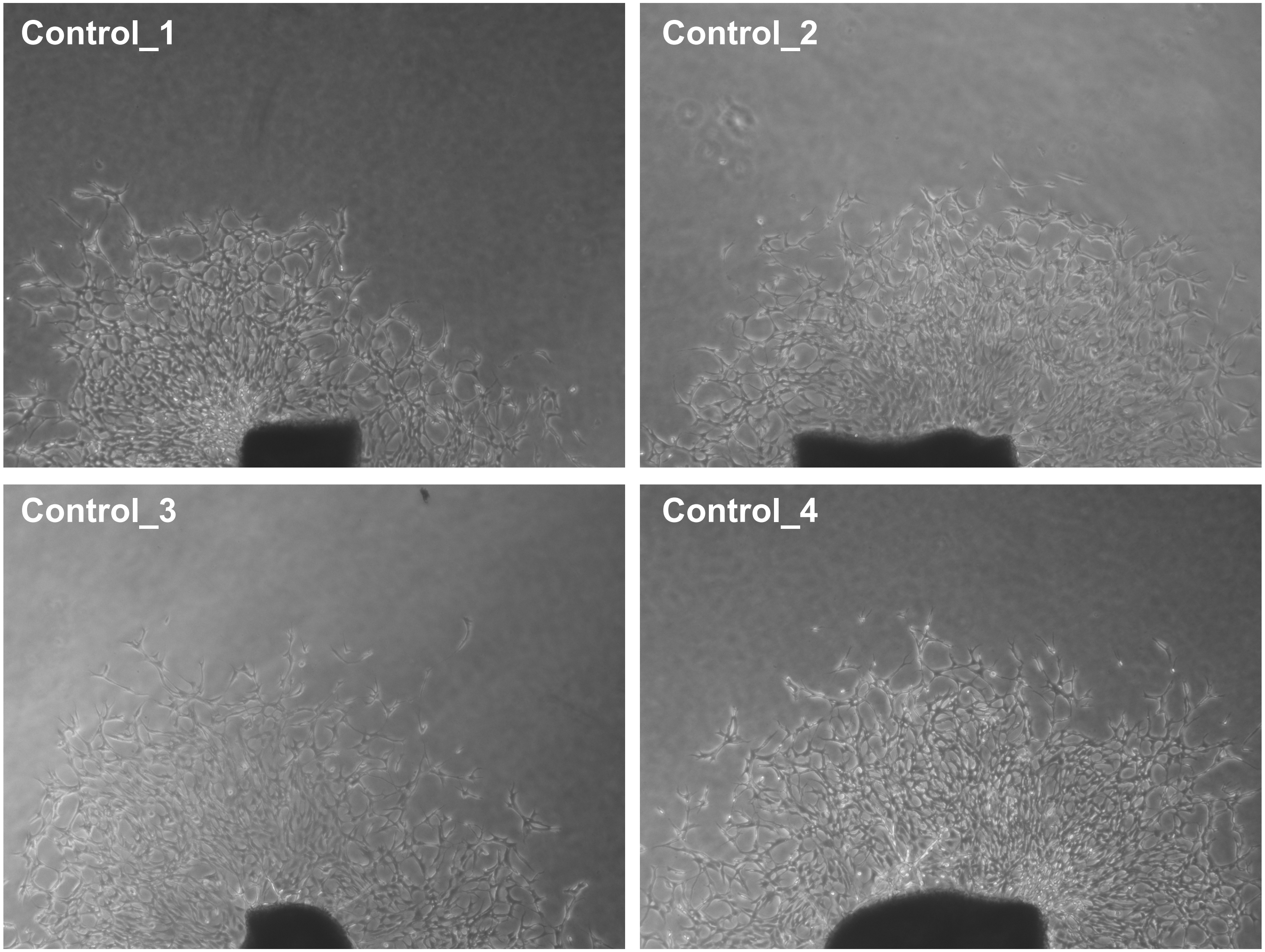

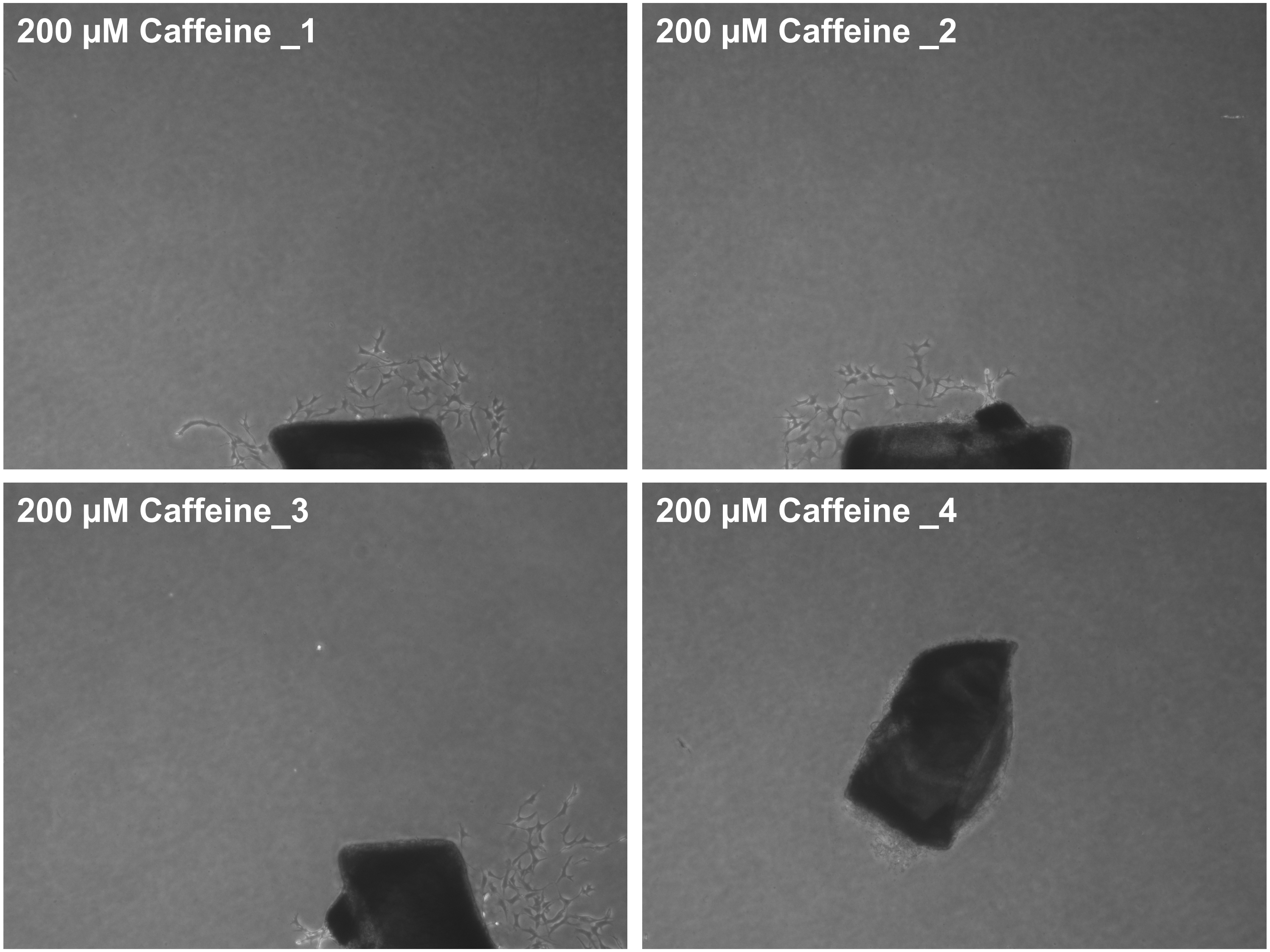
200 μM Caffeine _1
200 μM Caffeine _2
200 μM Caffeine_3
200 μM Caffeine _4

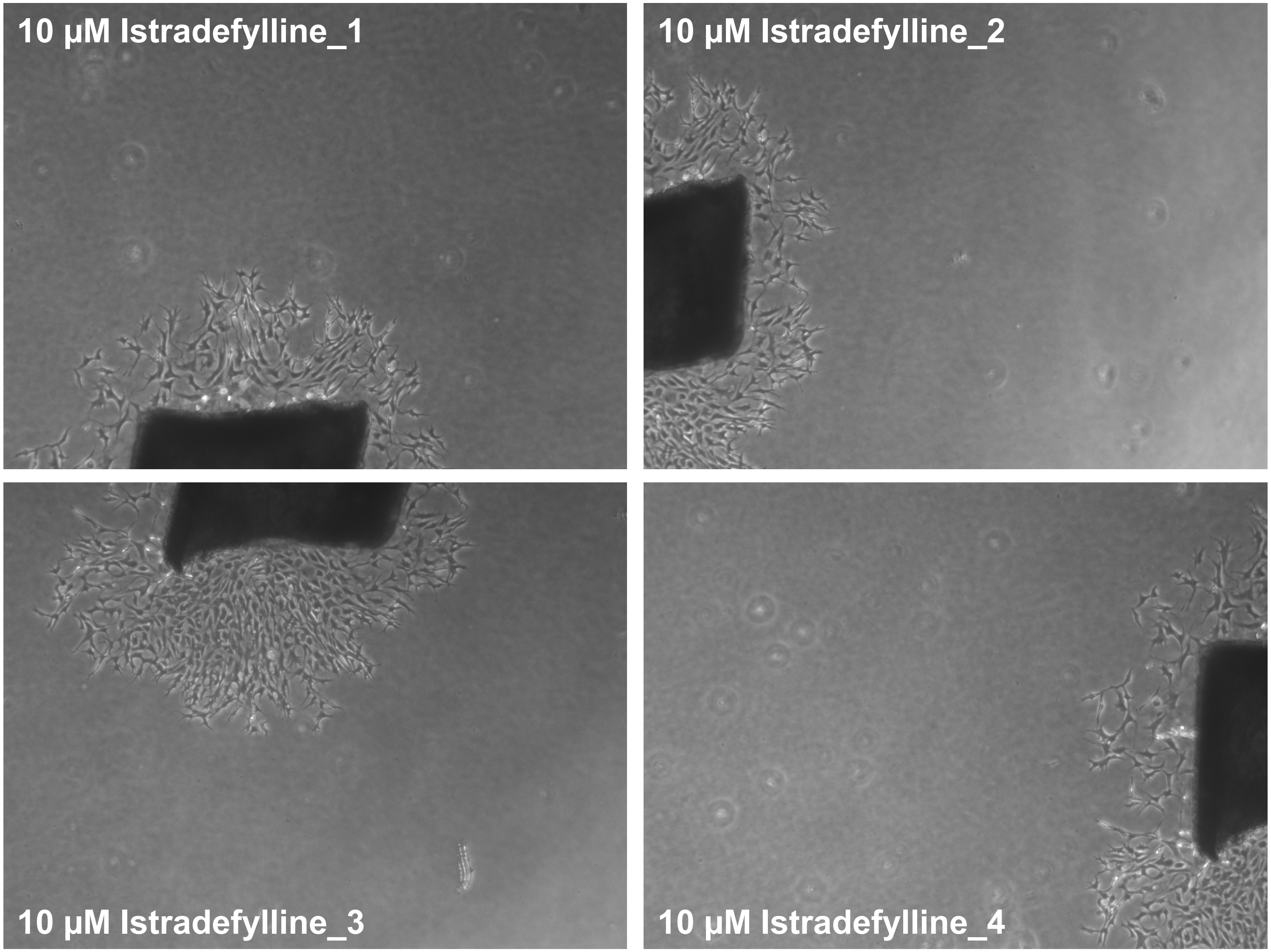
10 μM Istradefylline_1
10 μM Istradefylline_2
10 μM Istradefylline_3
10 μM Istradefylline_4
